# Raman Hyperspectral Imaging for Detection of Watermelon Seeds Infected with *Acidovorax citrulli*

**DOI:** 10.3390/s17102188

**Published:** 2017-09-23

**Authors:** Hoonsoo Lee, Moon S. Kim, Jianwei Qin, Eunsoo Park, Yu-Rim Song, Chang-Sik Oh, Byoung-Kwan Cho

**Affiliations:** 1 Environmental Microbial and Food Safety Laboratory, Agricultural Research Service, U.S. Department of Agriculture, Powder Mill Rd. Bldg. 303, BARC-East, Beltsville, MD 20705, USA; hoonsoo.lee@ars.usda.gov (H.L.); moon.kim@ars.usda.gov (M.S.K.); jianwei.qin@ars.usda.gov (J.Q.); 2 Department of Biosystems Machinery Engineering, College of Agricultural and Life Science, Chungnam National University, 99 Daehak-ro, Yuseong-gu, Daejeon 34134, Korea; besoo12@cnu.ac.kr; 3 Department of Horticultural Biotechnology and Institute of Life Science and Resources, Kyung Hee University, Yongin 441-701, Korea; yulimy@khu.ac.kr (Y.-R.S.); co35@khu.ac.kr (C.-S.O.)

**Keywords:** Raman hyperspectral imaging, spectral analysis, image processing, seed quality

## Abstract

The bacterial infection of seeds is one of the most important quality factors affecting yield. Conventional detection methods for bacteria-infected seeds, such as biological, serological, and molecular tests, are not feasible since they require expensive equipment, and furthermore, the testing processes are also time-consuming. In this study, we use the Raman hyperspectral imaging technique to distinguish bacteria-infected seeds from healthy seeds as a rapid, accurate, and non-destructive detection tool. We utilize Raman hyperspectral imaging data in the spectral range of 400–1800 cm^−1^ to determine the optimal band-ratio for the discrimination of watermelon seeds infected by the bacteria *Acidovorax citrulli* using ANOVA. Two bands at 1076.8 cm^−1^ and 437 cm^−1^ are selected as the optimal Raman peaks for the detection of bacteria-infected seeds. The results demonstrate that the Raman hyperspectral imaging technique has a good potential for the detection of bacteria-infected watermelon seeds and that it could form a suitable alternative to conventional methods.

## 1. Introduction

The watermelon is one of the most popular vegetables in the world, with a global production of more than 1.1 billion tons per year [1]. However, several pathogens such as *Acidovorax citrulli* and the cucumber green mottle mosaic virus (CGMMV), which can affect the growth of watermelons, have seriously affected its worldwide production [2,3,4]. Since infections caused by such pathogens are difficult to treat, it is important to detect them early and prevent the spread of diseases [5]. In addition, these pathogens can be masked in the seeds.

The common methods for the detection of pathogens in seeds have mainly involved semi-selective media, seedling grow-out, enzyme-linked immunosorbent assay (ELISA), and reverse transcription polymerase chain reaction (RT-PCR) [6,7]. The inherent drawbacks of these methods include the impossibility of reusing the sample, a diagnosis time of up to one day, and the requirement of skilled professionals to apply the methods. Therefore, it is necessary to develop new technologies to overcome these disadvantages.

In order to detect morphological and structural differences in plants, various techniques have been attempted, such as optical coherence tomography (OCT) [8,9,10,11], X-ray tomography [12,13,14], positron emission tomography (PET) [15,16,17], magnetic resonance imaging (MRI) [18,19,20], and ultrasound [21,22,23]. However, the use of these techniques has been limited to research on the structural analysis of plants.

Meanwhile, vibrational spectroscopy has been applied in various fields such as medicine, pharmacy, pathology, food, and agriculture [24,25]. In agriculture, the visible and near-infrared wavelengths (between 400 nm and 2500 nm) have been mainly employed to analyze fungi, insects, bruises, and defects in agricultural products [26,27]. However, the combined overtone absorption bands in the near-infrared region result in widely overlapping spectra, with near-infrared spectroscopy offering a detection limit of about 0.1%.

In this context, Raman spectroscopy offers the advantages of providing simultaneous physical and chemical information on an object of interest [28]. The Raman scattering phenomenon was first discovered in 1928 by Sir C.V. Raman [29], and Raman spectroscopy involves the analysis of Raman scattering, which is observed in samples excited by a strong light source [30]. However, there were initially very few applications of Raman spectroscopy because the technologies for detecting Raman-scattering effects and strong light sources were not sufficiently developed. With the development of such technologies, Raman spectroscopy began to be applied extensively [31]. Raman spectroscopy provides detailed information on molecular vibrations, and it has been found to offer extremely high detection sensitivity in various applications such as the quality assessment of meat and fish [32], detection of bacteria [33], prediction of components of agricultural products [34,35], and classification of oil and fat [36]. However, the application of spectroscopic techniques with traditional single-point laser instruments is limited to objects that are considerably larger than the probed area or are non-uniformly distributed [37].

The hyperspectral imaging technique has emerged as a novel technology that combines spectroscopy and imaging [26]. This technique provides spectral information along with spatial information. Hyperspectral image data have been efficiently used in the discrimination of agricultural products with uneven surfaces. In this regard, Delwiche and Kim detected scab (*Fusarium* head blight) in wheat using discriminant analysis [38], while Zhang et al. used visible near-infrared (Vis/NIR) hyperspectral imaging to detect late blight disease in tomatoes [39]. Grapefruit, vidalia sweet onions, and sweet orange have also been subject to hyperspectral analysis to detect citrus canker, blue mold, and sour skin disease, respectively [40,41].

Recently, Raman hyperspectral imaging has been applied in the detection of adulteration and additives in food. A macro-scale Raman hyperspectral imaging system has been developed and successfully applied to detect various chemical adulterants mixed in samples, such as melamine in milk powder, benzoyl peroxide in flour, and maleic anhydride in starch [42,43,44,45]. However, there have been no studies thus far on the application of Raman hyperspectral imaging in detecting bacteria-infected watermelon seeds.

The purpose of this study was to investigate the feasibility of using Raman hyperspectral imaging for the detection of watermelon seeds infected with *Acidovorax citrulli*. Our specific objectives were to: (1) establish a Raman hyperspectral imaging system; (2) identify the significant Raman peaks based on the F-values of an analysis of variance (ANOVA); (3) visualize changes in infected and healthy seeds; and (4) investigate the potential of Raman hyperspectral imaging for discriminating bacteria-infected seeds from healthy ones.

## 2. Materials and Methods

### 2.1. Bacteria-Infected Watermelon Seeds

The watermelon seeds infected with the bacteria *Acidovorax citrulli* were acquired by using the artificial inoculation method. The seeds were immersed in a medium containing high concentrations (108 CFU/mL) of bacteria. The bacterial strains were allowed to penetrate the watermelon seeds by using a vacuum. In order to remove bacteria from the seed surface, the surface was washed twice using distilled water. Then samples were naturally dried under the ambient condition for 48 h and measured by the Raman hyperspectral imaging system. After acquiring images, each sample was stored in a separate container to measure the bacteria count by the determination of a viable count. Each sampling was carried out through grinding, centrifugation, and culture. The cultivar of watermelon seeds was “speed plus”, obtained from the N Company in South Korea. In this study, 48 watermelon seeds were used to acquire the Raman hyperspectral image.

### 2.2. Raman Hyperspectral Imaging System

#### 2.2.1. System Design, Operation, and Software

**Figure 1:**
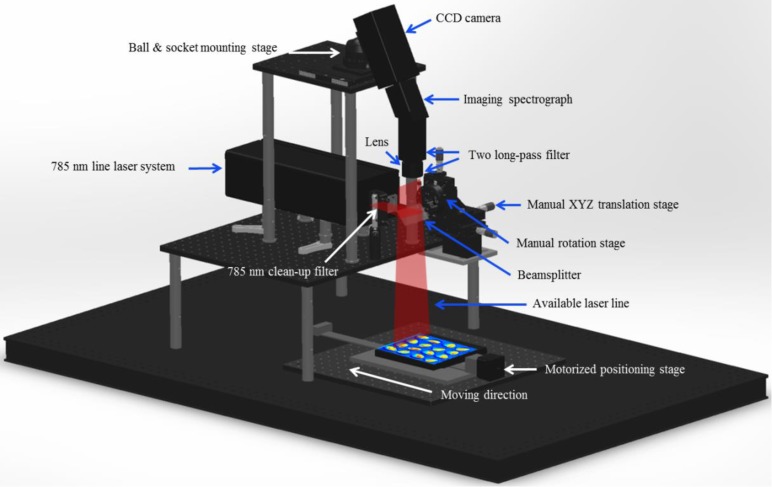
Schematic diagram of the main components of the Raman hyperspectral imaging system.

Figure 1 shows the schematic of the Raman hyperspectral imaging system used in the study, which consists of a CCD (Charge Coupled Device) camera, spectrograph, laser module, beam splitter, and moveable stage. It is necessary to use a high-performance CCD camera for imaging because the Raman signal involves a small fraction of the photons scattered by excitation (approximately 1 in 10 million). The Andor iKon-M 934 series camera (iKon-M 934, Andor, Concord, WI, USA) was used to collect the Raman-scattering data. The CCD had an area array of 1024 × 1024 pixels with a spectral response greater than 90% at 800 nm. The cooling temperature was set to −65 °C to reduce the dark noise of the CCD. The dynamic range of the camera sensor was 16 bits, and the camera was fixed along the vertical direction with the use of a ball-type regulator.

Here, we point out that it is also important to use a high-power laser system along with a high-performance camera to obtain a strong Raman signal; therefore, we used a spin-exchange optical-pumping laser system. This system consists of two spatially combined 785-nm laser heads mounted on a water-cooled cold plate, a laser-diode power supply, a system chiller, a collimating lens, and a clean-up filter. The laser beams generated by the two spatially combined laser heads pass through the collimating lens, resulting in a line laser. In the study, the laser head was positioned along the horizontal direction. A 785-nm laser clean-up filter (Semrock, LL01-785-25, Rochester, NY, USA) was set in front of the collimating lens to improve the signal-to-noise ratio of the laser system. The maximum output of the system was 30 W. A Raman imaging spectrograph (ImSpector R10E, Specim, Oulu, Finland) was used to disperse Raman scattering from the sample light, with the spectrograph being attached to the CCD camera. A C-mount lens (Schneider Optics, XENOPLAN 1.4/23 mm compact lens, Hauppauge, NY, USA) was mounted on the imaging spectrograph for adjusting the aperture and focus. Two 785-nm long-pass filters (Semrock, LP02-785RE-25, Rochester, NY, USA) were positioned between the lens and the spectrograph to eliminate Rayleigh scattering from the sample.

**Figure 2:**
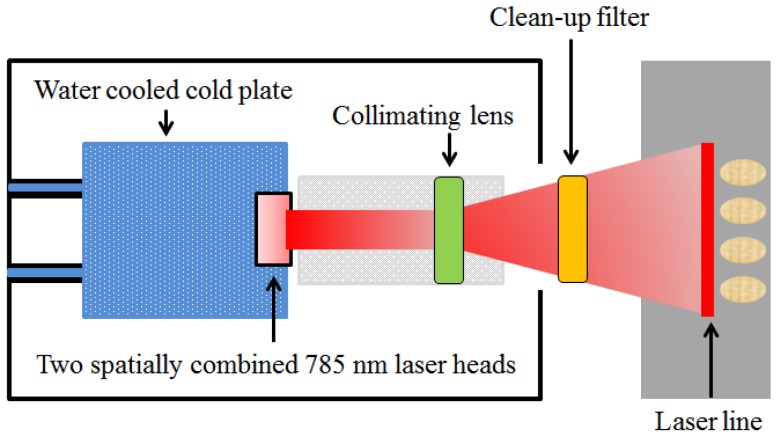
Line laser system used as the excitation source of the Raman hyperspectral imaging system.

Figure 2 shows the line laser generator used as the excitation source of the Raman hyperspectral system. In this system, first, a strong laser signal generated from the laser module reaches a custom-designed dichroic beam splitter, which reflects the incident laser at 45° while efficiently allowing the longer Raman-shifted wavenumbers to pass through. An advantage of this approach is that the position and size of the sample do not significantly influence the results. In our study, the Raman hyperspectral images were acquired by advancing a computer-controlled stage (Velmex, Model XN10-0180-M02-21, Bloomfield, NY, USA) in the direction perpendicular to the camera line images. The software to control the camera and step motor was developed with MATLAB (version 7.14, Mathworks, Natick, MA, USA). The software could adjust the variables of the camera (cooling temperature, exposure time, and pre-amplifier gain and accumulation) and motor (size of step, number of scans) for optimum Raman signal acquisition.

#### 2.2.2. System Calibration

The purpose of the spectral calibration of the Raman imaging system is to define the relative Raman-shift position corresponding to the pixel position of the CCD sensor array. The well-known chemical components for Raman calibration, naphthalene, 4-cetamidophenol and polystyrene, were used for spectral calibration of the Raman hyperspectral imaging system. These chemicals were scanned using the Raman hyperspectral imaging system, and the corresponding Raman spectrum was extracted from the Raman image data. An average of 100 pixels were extracted for each chemical, and 15 Raman peaks were selected for determining the relationship between the 15 standard wavenumbers and the pixels in the spectral region. A quadratic function was used for regression analysis. The regression coefficient was subsequently determined as 0.9999, and the residual errors estimated using the quadratic equation were less than 0.12 cm^−1^. The Raman shift based on the quadratic regression model was calculated to range from −724.4 cm^−1^ to 2879.3 cm^−1^ (742.8 nm to 1014.2 nm), and the average wavenumber interval (spectral resolution) was 3.52 cm^−1^ (0.19 nm).

The spatial resolution was determined using the focal length of the lens and the distance between the lens and sample. When a lens of focal length 23 mm and working distance 370 mm was set, the spatial resolution for one pixel was calculated as 0.20 mm with the use of the standard US Air Force 1951 resolution grid chart (Edmund Scientific Co., Barrington, NJ, USA).

#### 2.2.3. Image Acquisition and Spectral Extraction

In order to obtain the optimal Raman scattering signal, watermelon seeds were positioned such that they were excited uniformly by the line laser. The moving distance was set as 0.20 mm to acquire Raman images with the same spatial resolution. The Raman image was obtained without binning for pixels along the horizontal and vertical directions. In order to obtain an optimal Raman signal without damaging the seeds, the laser power and CCD exposure time were set to 5 W and 1 s, respectively. Two hundred and fifty lines were gathered from seed samples, resulting in a 1024 × 250 × 1024 hypercube (250 scans and 1024 bands). A dark image was also acquired with a cap covering the lens to subtract from the original Raman hyperspectral image data. After image acquisition and calibration (subtraction of the dark image), the region of interest (ROI) was extracted using the Raman hyperspectral image data. The image at 886.4 cm^−1^ was selected for eliminating the background, and the Raman spectra of the seeds were easily separated from the background generated by the sample plate. Approximately 800 spectra were acquired from the watermelon seeds. Image acquisition, calibration, and spectral extraction were performed using MATLAB (Mathworks, Natick, MA, USA).

### 2.3. Baseline Correction and Data Analysis

**Figure 3:**
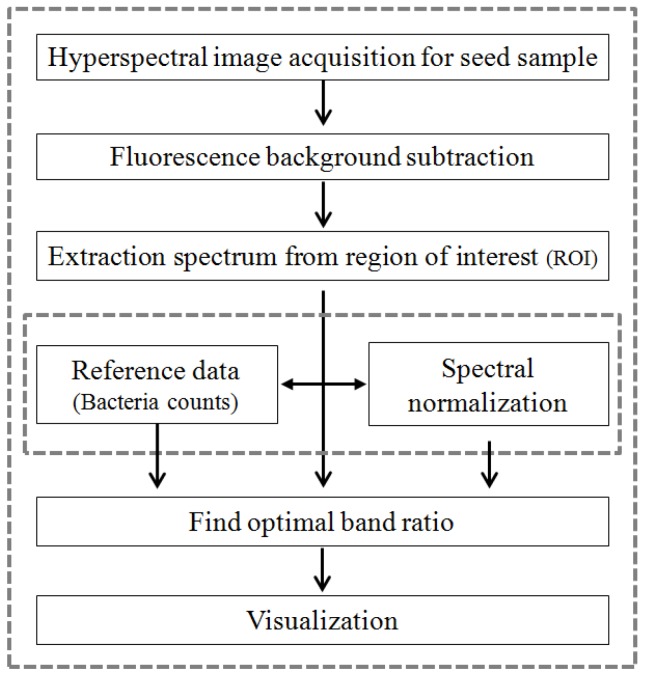
Flowchart of the development procedures for analyzing hyperspectral images and for displaying the difference between infected and healthy seed samples.

The flowchart of the procedures for analyzing the hyperspectral images and for displaying the difference between infected and healthy seed samples is shown in Figure 3. The first step in the analysis of the acquired Raman hyperspectral image data is to remove the fluorescence signal. Here, we recall that the Raman scattering signal was generated using a strong laser light source. Furthermore, biological materials such as agricultural products may emit strong fluorescence signals that mask the characteristic Raman scattering signal, a problem that has been considered a challenge for Raman spectroscopy [46]. In the present study, a widely used polynomial fitting method was employed to analyze the Raman spectral data and to correct for fluorescence, because this method is efficient and simple [47,48]. Polynomial fitting involves determining the polynomial of the proper order for obtaining a baseline through iterative calculation. Polynomials of various orders such as 4, 5, 8, 12, and 16 were tested for fitting the spectral data based on previous studies that used a 5th-order polynomial for soybeans [28] and an 8th-order polynomial for lycopene in tomatoes [46]. Finally, a 16th-order polynomial equation and the 100th iteration were employed to create the fluorescence-correction baseline, because its prediction was the best among all polynomials.

The Raman spectra for ROIs were extracted from the hyperspectral image data. Next, reference data for bacterial infection of seed samples and Raman spectra modified with the polynomial fitting method were utilized to determine the optimal band-ratio combination that could discriminate infected seeds from healthy seeds. An ANOVA was used to determine the optimal band-ratio combination. An ANOVA is one of the most robust and frequently used statistical methods to analyze the differences between groups [4]. It was used to determine the optimal wavebands combination for the discrimination between infected and healthy seeds, and approximately 2400 spectra of infected seed and 24,000 spectra of healthy seed were used in this study. The *F*-values of a one-way ANOVA were used to find the wavelengths representing statistically significant differences for the two groups. The 80% of spectral data and remaining 20% spectral data were employed for calibration and validation, respectively.

## 3. Results and Discussion

### 3.1. Spectral Analysis

**Figure 4:**
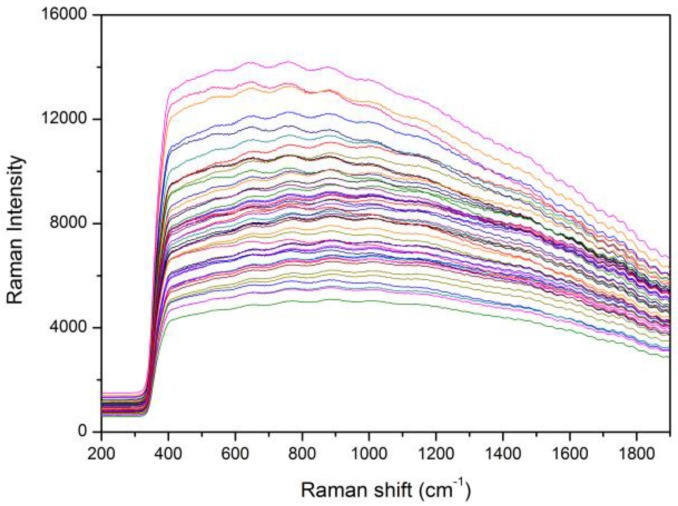
Original Raman spectra of bacteria-infected and healthy watermelon seeds.

**Figure 5:**
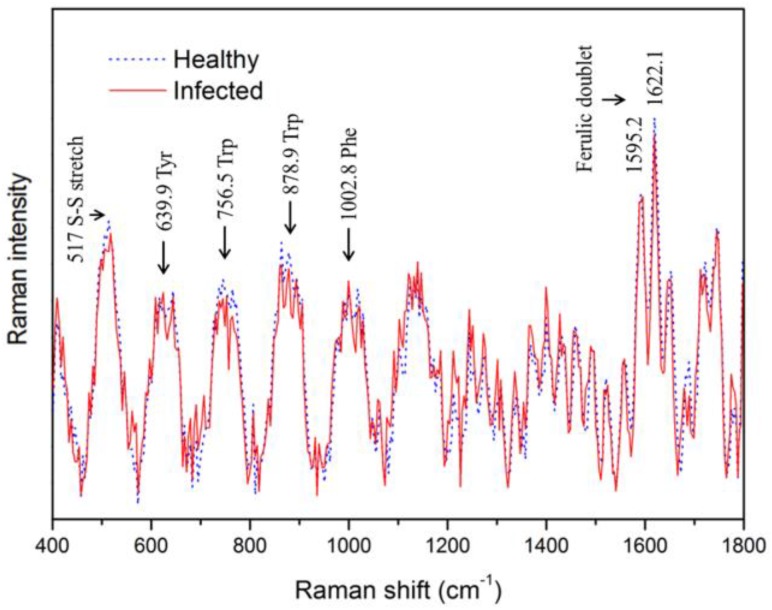
Mean corrected Raman spectra of bacteria-infected and healthy watermelon seeds obtained by removing the fluorescence signal using a polynomial equation.

Figure 4 shows the original Raman spectra of healthy and bacteria-infected watermelon seeds in the wavenumber range of 200–1900 cm^−1^. Among the 48 inoculated samples in this study, we confirmed 4 infected and 44 healthy samples. The healthy and bacteria-infected watermelon seeds did not show any noticeable Raman peaks. Furthermore, there was a large intensity variation due to the fluorescence background. In order to remove the fluorescence signal, we used a 16th-order polynomial equation, as mentioned previously. Figure 5 shows the clear Raman mean spectra for infected and healthy watermelon seeds; the two spectral patterns are similar. From the figure, we note that prominent Raman peaks appear at 517 cm^−1^ (S–S stretching), 639.9 cm^−1^ (tyrosine), 756.5 cm^−1^ (tryptophane), 878.9 cm^−1^ (tryptophane), 1002.8 cm^−1^ (phenylalanine), 1595.2 cm^−1^ (ferulic acid), and 1622.1 cm^−1^ (ferulic acid). However, the two sets (healthy and infected seeds) show no significant difference.

### 3.2. ANOVA for Classification of Bacteria-Infected and Healthy Seeds

**Figure 6:**
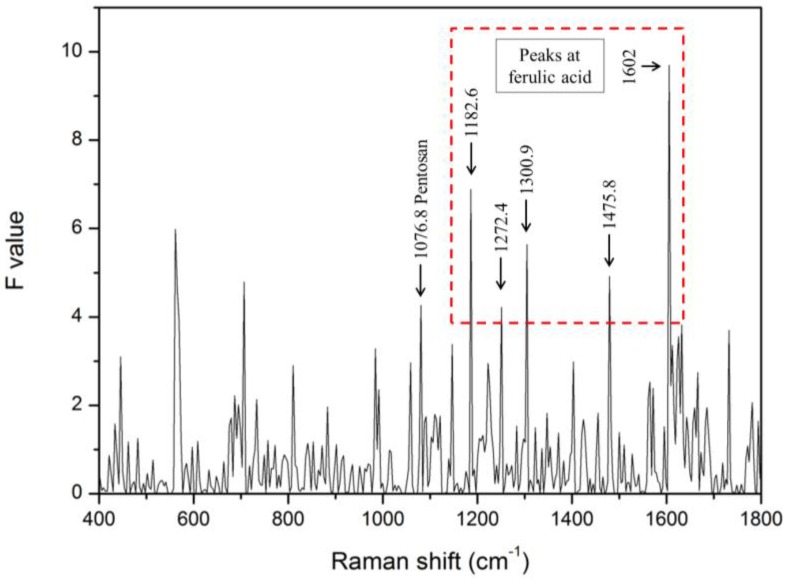
*F*-values of each Raman shift between infected and healthy seeds determined using ANOVA.

The ANOVA test was used to determine Raman peaks that present a significant difference between the infected and healthy seeds. The *F*-values of each Raman spectrum for infected and healthy seeds are shown in Figure 6. Twelve statistically significant Raman peaks were selected through ANOVA (*p* < 0.01). Among these peaks, those at 1076.8 cm^−1^, 1182.6 cm^−1^, 1272.4 cm^−1^, 1300.9 cm^−1^, 1475.8 cm^−1^, and 1602 cm^−1^ were almost identical to the peaks corresponding to pentosan and ferulic acid, as reported in previous experimental studies [49].

Pentosan (arabinoxylan) and ferulic acid are well known as cell-wall constituents. In particular, pentosan plays the role of supporting the cell walls through the hydroxyl-cinnamate bond, and it consists of an acetyl group and a feruloyl group joined together [50]. Indeed, ferulic acid is one of the phenolic compounds found in infected plants [51].

The range of pentosan content is approximately 2–8% in seeds with variety, and the endosperm and aleurone layer (except for rice and barley) contain 60–70% of pentosan. In summary, this result is assumed to be due to the difference in the pentosan (arabinoxylan) and ferulic acid content in watermelon seeds [50]. In other words, this phenomenon can be considered as due to changes resulting from the pathogenic infection of seeds.

### 3.3. Visualization of Bacteria-Infected Seeds

**Figure 7:**
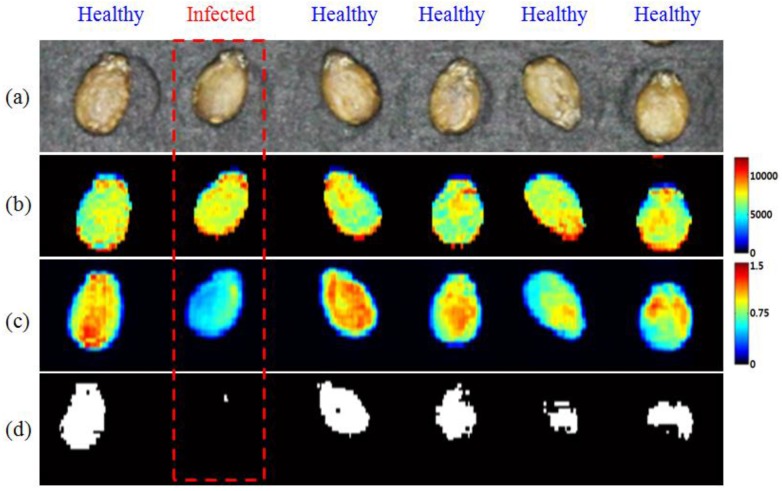
Comparison of the resultant images obtained by using the Raman hyperspectral image; (**a**) original picture of seed samples, (**b**) representative Raman images at 1076.8 cm^−1^, (**c**) a band-ratio image (1076.8 cm^−1^/437 cm^−1^), (**d**) a binary band-ratio image with a threshold value of 1.03.

Figure 7a shows the color images of the watermelon seed samples. The second seed from the left in the box indicated by the red dotted line was identified as the infected one among six seeds. First, the ANOVA test was used to determine the single-Raman-shift positions. Figure 7b shows the single-Raman-shift images at 1076.8 cm^−1^. A 1076.8-cm^−1^ Raman image was selected as being able to best discriminate between the two sets of seeds. However, this image does not show a clear difference between the two sets. This phenomenon with Raman and fluorescence signals commonly occurs when a strong light such as a laser is irradiated onto plants. The result indicates that it is difficult to detect infected seeds using only a single Raman image. Hence, ANOVA was performed to determine the optimal Raman-shift band-ratio for use as the discriminant between infected and healthy seeds.

**Figure 8:**
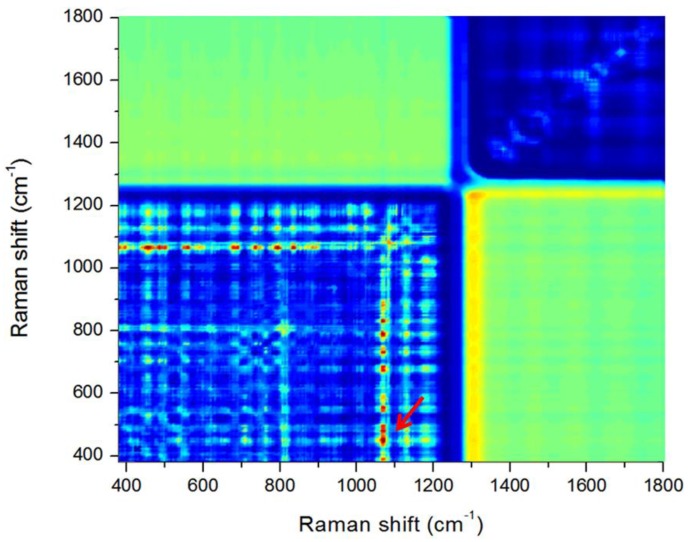
*F*-values for wavenumber pairs used for determining the optimal Raman shift band-ratio between bacteria-infected and healthy watermelon seeds.

The *F*-values of ANOVA for all the Raman-shift band-ratio combinations for the infected and healthy seeds were calculated in the range 400–1800 cm^−1^ to determine the optimal band-ratio. A large *F*-value between the two groups indicated a statistically significant separation. Figure 8 shows the resultant contour plot of *F*-values obtained from ANOVA for distinguishing between infected and healthy seeds. The plot shows multiple clusters of band-ratio pair regions with relatively high *F*-values (red color). The ratio of the two bands at 1076.8 cm^−1^ and 437 cm^−1^ provided the best result for separating infected and healthy seeds. However, the Raman peaks at 1076.8 cm^−1^ and 437 cm^−1^ did not show any remarkable features when compared with the main peaks in Figure 5. We determined that the features do not appear due to the relatively strong signals of the Raman peaks around these two values.

As shown in Figure 7c, an infected seed has relatively fewer red pixels. Figure 7d shows a binary image obtained with a threshold value of 1.03. This image demonstrates that the number of pixels for healthy seeds was significantly higher than that for infected seeds. The band-ratio combinations indicate that the composition ratio of starch (437 cm^−1^) and pentosan (1076.8 cm^−1^) in the two groups of seeds is different [52].

The classification accuracy for a total of 48 samples was evaluated using the average Raman spectrum of each sample. The average band-ratio values for healthy and infected seeds were 1.22 ± 0.21 and 0.96 ± 0.11, respectively. When applied as a threshold value of 1.03, 3 of 4 infected seeds (75%) and 38 of 44 healthy seeds (86%) were detected as true positives by band-ratio combinations, respectively. Of the 48 seed samples that were not known to be infected, 4 seeds were finally identified as infected seeds. The number of samples was insufficient to compare the sorting performance of the 4 infected seeds with that of the 44 healthy seeds. However, the thousands of units of spectral information obtained from each group provides sufficient information to identify inter-group differences.

## 4. Conclusions

This study was carried out to evaluate the feasibility of using a Raman hyperspectral imaging system for discriminating between bacteria-infected and healthy watermelon seeds. The results indicated that it was possible to employ this non-destructive technique to detect changes due to the bacterial infection of watermelon seeds. The ANOVA method was suitable for determining the optimal Raman band-ratio (1076.8 cm^−1^/437 cm^−1^) for the detection of bacteria-infected watermelon seeds. In addition, the baseline correction method for removing the fluorescence signal included in the original data could be successfully applied to analyze the characteristics between infected and healthy seeds. Our results indicate that Raman hyperspectral imaging can detect bacteria-infected seeds as well as aid in investigating changes caused by infection.
